# Diagnostic value of gut microbiota profiling and circulating biomarkers to predict post-stroke infection in acute ischemic stroke

**DOI:** 10.3389/fmed.2025.1702025

**Published:** 2026-04-28

**Authors:** Weny Rinawati, Aryati Aryati, Abdulloh Machin, Stefan Kiechl, Gregor Broessner

**Affiliations:** 1 Doctoral Program in Medical Science, Faculty of Medicine, Universitas Airlangga, Surabaya, Indonesia; 2 Laboratory and Blood Bank Unit, National Brain Center Hospital Mahar Mardjono, Jakarta, Indonesia; 3 Department of Clinical Pathology, Faculty of Medicine, Universitas Airlangga, Surabaya, Indonesia; 4 Dr. Soetomo General Academic Hospital, Surabaya, Indonesia; 5 Department of Neurology, Faculty of Medicine, Universitas Airlangga, Surabaya, Indonesia; 6 Airlangga University Hospital, Surabaya, Indonesia; 7 Department of Neurology, Medical University of Innsbruck, Innsbruck, Austria; 8 VASCage - Center for Clinical Stroke Research, Innsbruck, Austria

**Keywords:** acute ischemic stroke, post-stroke infection, biomarkers, gut microbiota, diagnostic accuracy

## Abstract

**Background:**

Post-stroke infection (PSI), particularly pneumonia and urinary tract infection, is a common and serious complication after acute ischemic stroke (AIS). Current diagnostic biomarkers provide limited accuracy when used in isolation. This study aimed to evaluate the diagnostic value of circulating biomarkers and gut microbiota profiling, both individually and in combination, to predict PSI in AIS patients.

**Methods:**

We conducted a prospective observational study at Prof. Dr. dr. Mahar Mardjono National Brain Center Hospital, Jakarta. A total of 80 AIS patients admitted within 24 h of onset were enrolled and followed for 7 days to assess PSI. Blood samples were analyzed for NMDAR, butyrate, TMAO, RANKL, iFABP, and LPS. Stool samples were collected for 16S rRNA sequencing. Diagnostic performance was evaluated using ROC curves, with AUC, sensitivity, and specificity calculated. Multivariate logistic regression models were constructed to assess independent predictors and combined diagnostic accuracy.

**Results:**

PSI occurred in 37/80 patients (46.3%). NMDAR showed the highest diagnostic performance (AUC 0.911; sensitivity 86.5%; specificity 90.7), followed by iFABP (AUC 0.894), LPS (AUC 0.896), RANKL (AUC 0.881), butyrate (AUC 0.866), and TMAO (AUC 0.865). Gut microbiota analysis revealed reduced evenness and dominance imbalance in infected patients, with enrichment of pathogenic taxa (*Escherichia coli, Salmonella enterica*) and depletion of SCFA-producing commensals (*Faecalibacterium prausnitzii, Roseburia intestinalis*). Multivariate models integrating microbiota features and biomarkers improved predictive accuracy compared with single-domain approaches.

**Conclusion:**

Integrating circulating biomarkers with gut microbiota profiling significantly enhances early prediction of PSI in AIS. These findings highlight the role of the gut–brain–immune axis in post-stroke complications and support combined biomarker–microbiota models for risk stratification and preventive strategies.

## Introduction

Acute ischemic stroke (AIS) remains a leading global cause of disability and mortality (1). One critical complication following AIS is post-stroke infection (PSI)—commonly presenting as pneumonia or urinary tract infection—which worsens functional outcomes, prolongs hospitalization, increases costs, and elevates mortality risk. Although single circulating biomarkers (e.g., NMDAR antibodies, butyrate, TMAO, RANKL, iFABP, and LPS) have been explored, their diagnostic performance (AUC, sensitivity, specificity) is variable and often limited in isolation. Recent syntheses specific to stroke-associated pneumonia (SAP) show that inflammatory biomarkers—particularly procalcitonin (PCT)—can aid early identification, but optimal accuracy likely requires integration with clinical or biological features (1, 2).

Concurrently, growing evidence underscores the role of the gut microbiota in post-stroke immune modulation and PSI risk. Lower abundance of short-chain fatty acid (SCFA)–producing taxa after AIS has been linked to SAP, and integrated models that combine microbiota features with clinical scores outperform single-domain predictors (e.g., AUC ≈ 0.81 vs. ≈ 0.70 for differential genera alone) (2). Moreover, microbial dysbiosis characterized by depletion of Roseburia and enrichment of opportunists independently predicts SAP among AIS patients, strengthening the rationale for incorporating microbiota signatures into risk stratification (3).

Beyond microbiota composition, microbiota-derived and barrier-injury markers are mechanistically plausible PSI predictors in AIS. Circulating LPS levels rise over time after AIS and are associated with worse neurological outcomes, supporting the concept that endotoxemia and impaired host defenses contribute to post-stroke infectious complications (4). Likewise, markers of intestinal barrier injury such as intestinal fatty acid–binding protein (iFABP) are elevated in AIS and reflect mucosal damage and potential bacterial translocation—key pathways linking dysbiosis to infection susceptibility (5).

Despite these advances, studies that combine gut microbiota profiling with circulating biomarkers (e.g., PCT/CRP/IL-6, iFABP, LPS; and disease-relevant metabolites such as TMAO) remain scarce in PSI prediction. Evidence indicates that higher TMAO relates to stroke onset, severity, and outcomes, suggesting added value when integrated with infection-or barrier-focused markers in multivariate models (6). Traditional inflammatory biomarkers such as C-reactive protein (CRP), PCT, and white blood cell count (WBC) are routinely used for early infection screening in post-stroke settings; however, they lack specificity and are often confounded by post-stroke systemic inflammation rather than true infection. To address this diagnostic gap, we focused on mechanistically relevant biomarkers that reflect distinct components of the gut–brain–immune axis: NMDAR antibodies (neuroimmune activation), RANKL (inflammatory signaling and tissue remodeling), iFABP (intestinal barrier disruption), LPS (bacterial translocation and endotoxemia), TMAO (microbial metabolite linked to vascular inflammation), and butyrate (a short-chain fatty acid critical for mucosal integrity and immune modulation). Based on this rationale, we hypothesized that integrating gut microbiota profiles with these circulating biomarkers would improve diagnostic accuracy for post-stroke infection compared with single biomarker approaches. Accordingly, this study will (i) quantify the diagnostic accuracy (AUC, sensitivity, specificity) of individual biomarkers (NMDAR, butyrate, TMAO, RANKL, iFABP, LPS), (ii) assess the independent predictive value of gut microbiota profiles, and (iii) develop and validate combined multivariate models integrating microbiota and circulating biomarkers to enhance early PSI prediction in AIS.

## Methods

### Study design and population

This was a observational study conducted at Prof. Dr. dr. Mahar Mardjono National Brain Center Hospital, Jakarta, Indonesia between October 2023 and September 2024. Eligible participants were adult patients (≥18 years) with a confirmed diagnosis of acute ischemic stroke (AIS) based on clinical evaluation and neuroimaging (CT/MRI), admitted within 24 h of symptom onset. Patients with pre-existing infections, autoimmune disorders, malignancy, immunosuppressive therapy, or recent antibiotic/probiotic use (within the last 4 weeks) were excluded. All data presented in this manuscript were derived from the same prospective AIS cohort described in our earlier publication, and this study represents a prespecified sub-analysis focusing on gut microbiota and endothelial biomarkers.

### Sample size

Sample size was calculated using a formula for diagnostic test studies, targeting an expected improvement in AUC of at least 0.10 between the combined (microbiota + biomarker) model and biomarker-only model. The calculation was based on the method described by Obuchowski (1994) for comparing correlated ROC curves, assuming an AUC0 = 0.80, AUC1 = 0.90, α = 0.05, β = 0.20, and equal group allocation. Using this approach, a minimum of 72 subjects was required; we recruited 80 patients to ensure adequate power for subgroup and multivariate analyses.

### Data collection and definitions

Patients with acute ischemic stroke (AIS) who met the inclusion criteria and did not meet the exclusion criteria were recruited for this study. Upon admission, baseline demographic and clinical data were recorded, including age, sex, vascular risk factors, comorbidities, and stroke characteristics. Stroke severity was assessed using the National Institutes of Health Stroke Scale (NIHSS).

Specimens were collected prior to antibiotic administration. Venous blood specimens were collected on the first day and within 3 days of treatment, while stool specimens were collected within 3 days of treatment. Venous blood specimens were collected using one 4 mL activator serum for NMDAR levels on the first day, and one 4 mL activator serum tube for NMDAR (second), butyrate, TMAO, RANKL, iFABP, and LPS levels, which were collected simultaneously with stool specimen collection. The collected specimens were then stored at −20 °C or −80 °C until simultaneous measurements were performed after all materials were collected. Blood and stool specimens were collected as paired samples. All blood and stool sampling occurred before antibiotic initiation to avoid confounding effects on microbiota composition and biomarker levels.

In addition, all patients underwent an initial procalcitonin test at baseline to confirm the absence of bacterial infection at study entry. A second procalcitonin test was performed either at the time of infection onset or on day 7 if no infection occurred. Patients were followed throughout hospitalization, and infection events during the acute phase (3–7 days) were recorded. Grouping into infectious and non-infectious categories was determined based on the second procalcitonin result.

PSI was defined according to international standards:

- Pneumonia and urinary tract infection (UTI): based on CDC criteria.
- Other systemic infections: according to Systemic Inflammatory Response Syndrome (SIRS) criteria.
- Sepsis: defined by Sepsis-3 criteria, as an increase in SOFA score ≥2 or qSOFA score ≥2 in the presence of suspected infection.

All figures were recreated in high resolution with standardized font sizes and color schemes. Duplicated content between PCA and PCoA plots was removed, and microbial labels were enlarged to ensure readability.

### Microbiota analysis

Fresh stool samples were obtained within 48 h of admission and stored at −80 °C. 16S rRNA sequencing targeting the V3–V4 region (Illumina platform) was performed. Bioinformatics analysis was conducted using QIIME2 pipeline. Alpha diversity (Shannon, Chao1 indices), beta diversity (Bray–Curtis dissimilarity), and differential taxonomic abundance (LEfSe analysis) were assessed. Specific taxa of interest included butyrate-producing genera (e.g., *Roseburia, Faecalibacterium*) and TMAO-producing bacteria. Because sequencing was restricted to the V3–V4 region of the 16S rRNA gene, species-level resolution was limited, and functional inference could not be fully achieved. LEfSe results were supplemented with FDR-adjusted *p*-values (Benjamini–Hochberg correction), and taxa with an FDR <0.10 were considered significant. The LDA score threshold of >2.0 was chosen based on standard LEfSe recommendations for medium-effect discriminant taxa.

### Biomarker measurement

Blood samples were collected at admission (<24 h after stroke onset) and analyzed for:

- NMDAR antibodies (ELISA)
- Butyrate levels (HPLC)
- Trimethylamine N-oxide (TMAO) (LC-MS/MS)
- Receptor activator of NF-κB ligand (RANKL) (ELISA)
- Intestinal fatty acid-binding protein (iFABP) (ELISA)
- Lipopolysaccharide (LPS) (Limulus Amebocyte Lysate assay).

All assays were performed in duplicate, and laboratory personnel were blinded to clinical outcomes.

### Outcomes

The primary outcome was the occurrence of PSI within 7 days of AIS onset. Secondary outcomes included individual infection types (SAP vs. UTI) and in-hospital mortality.

### Statistical analysis

Continuous variables were expressed as mean ± SD or median (IQR) and compared using Student's *t*-test or Mann–Whitney *U* test, depending on distribution. Categorical variables were analyzed with χ^2^ test or Fisher's exact test.

- Univariate logistic regression was used to explore predictors of PSI.
- Multivariate logistic regression was performed, including significant clinical variables and biomarker levels.
- Diagnostic performance was assessed using ROC curve analysis. The AUC, sensitivity, specificity, and optimal cut-off values were calculated for:

° Single biomarkers (NMDAR, Butyrate, TMAO, RANKL, iFABP, and LPS),
° Microbiota indices (Alpha Diversity, Beta Diversity, F/B and F/P Ratios, and LEfSe Analysis).

A two-sided *p* < 0.05 was considered statistically significant. All analyses were conducted using SPSS v26 and R software (pROC, vegan, ggplot2 packages). Variables with *p* < 0.10 in univariate analysis were entered into a stepwise backward logistic regression to construct the final multivariate model. To improve internal validity, ROC analyses were repeated using 1,000-sample bootstrap internal validation.

### Ethical considerations

The study was approved by the Institutional Review Board of the National Brain Center Hospital Prof. Dr. Mahar Mardjono, Jakarta (Approval No. DP.04.03/D.XXIII.9/132/2023, dated October 16, 2023). Written informed consent was obtained from all participants or their legal representatives.

## Result

This study involved 80 patients with acute ischemic stroke who were divided into two groups: the infection group and the non-infection group. The diagnosis of acute ischemic stroke (AIS) was established based on neurological examination and brain imaging using CT scan or MRI. Meanwhile, the diagnosis of bacterial infection was determined according to hospital protocols through a combination of clinical findings, laboratory test results, and microbiological cultures. Regarding secondary outcomes, stroke-associated pneumonia (SAP) accounted for 73.0% of infections, followed by urinary tract infection (21.6%) and sepsis (5.4%). In-hospital mortality was observed in three patients (3.8%), all belonging to the infection group. Patients with SAP had significantly higher NIHSS scores and lower microbiota evenness compared with non-infected patients (*p* < 0.05). Units for each biomarker were as follows: NMDAR (ng/mL), RANKL (pg/mL), iFABP (ng/mL), LPS (pg/mL), TMAO (ng/mL), and butyrate (μmol/L). The clinical relevance of these biomarkers—such as elevated NMDAR reflecting neuroimmune activation, RANKL indicating inflammatory tissue remodeling, iFABP signaling gut-barrier injury, and LPS indicating endotoxemia—is discussed in detail below. The characteristics of the study sample are presented in Table 1.

**Table 1:** Baseline characteristic.

Variable	Total (*n* = 80)		Infection (*n* = 37)		Non-infection (*n* = 43)		*p*-value
	*n*	(%)	*n*	(%)	*n*	(%)	
Age, mean (SD)	62.4 (11.2)		66.8 (10.4)		58.7 (10.8)		**0.003** ^*^
Age group, years							
<60	33	41.3	9	24.3	24	55.8	**0.005** ^*^
≥60	47	58.8	28	75.7	19	44.2	
Gender							
Male	47	58.8	17	45.9	30	69.8	**0.027** ^*^
Female	33	41.3	20	54.1	13	30.2	–
Body mass index (BMI, kg/m^2^)							
*Underweight* (<18.5)	2	2.5	2	5.4	–	–	**0.029** ^*^
Normal (18.5–22.9)	18	22.5	12	32.4	6	14.0	
*Overweight* (23.0–24.9)	16	20.0	6	16.2	10	23.3	
Obese (>25.0)	44	55.0	17	45.9	27	62.8	
Cardiovascular risk factors							
Hypertension	50	62.5	22	59.5	28	65.1	0.296
Diabetes mellitus	34	42.5	16	43.2	18	41.9	0.556
Dyslipidemia	59	73.8	30	81.1	29	67.4	**0.089**
Hyperuricaemia	14	17.5	8	21.6	6	14.0	0.372
Smoking	7	8.8	3	8.1	4	9.3	0.509
Diet (%)							
High sodium	33	41.3	17	45.9	16	37.2	0.362
High sugar	35	43.8	18	48.6	17	39.5	**0.148**
High fat	34	42.5	13	35.1	21	48.8	**0.112**
High purine	13	16.3	6	16.2	7	16.3	0.508
Low fiber	3	3.8	2	5.4	1	2.3	0.470
Ward type							
Intensive care	3	3.8	3	8.1	-	-	**0.057**
Non-intensive care	77	96.3	34	91.9	43	100	
Infarct location							
Single lesion	13	16.3	5	13.5	8	18.6	0.797
Multiple lesion	60	75.0	29	78.4	31	72.1	
**NIHSS score, median (Q1–Q3)**	4.6 (4.0–9.0)		5.0 (4.0–5.0)		4.0 (3.0–7.0)		0.077
Stroke severity at admission							
Mild stroke	40	50.0	15	40.5	25	58.1	**0.074**
Moderate stroke	33	41.3	17	45.9	16	37.2	
Moderate to severe stroke	7	8.8	5	13.5	2	4.7	
Severe stroke	–	–	–	–	–	–	
**Diysphagia**	17	21.3	11	29.7	6	14.0	0.41^*^
Medical devices							
Ventilator	1	1.3	1	2.7	–	–	0.278
Central venous catheter (CVC)	1	1.3	1	2.7	–	–	0.278
Nasogastric tube (NGT)	14	17.5	8	21.6	6	14.0	**0.183**
Urinary catheter	2	2.5	1	2.7	1	2.3	0.914
Therapies and procedures							
Total parenteral nutrition (TPN)	5	6.3	5	13.5	–	–	**0.013** ^*^
Blood transfusion	1	1.3	1	2.7	–	–	0.278
Tracheostomy	1	1.3	1	2.7	–	–	0.278
**White blood cell count**, **×10**^**9**^**/L**	9.9 (2.9)		9.6 (2.8)		9.9 (3)		0.096
>10.000	39	48.8	12	32.4	27	62.8	0.006^*^
5.000–10.000	41	51.2	25	67.6	16	37.2	
<5.000	–	–	–	–	–	–	
Rasio^4^							
**Platelet count**, **×10**^**9**^**/L**	292 (248–354)		284 (247–332)		296 (250–357)		0.761
>400.00	13	16.5	8	22.2	5	11.6	**0.169**
150.00–400.00	67	83.8	29	78.4	38	88.4	
<150.00	–	–	–	–	–	–	
Procalcitonin, median (Q1–Q3), ng/mL							
First measurement	0.03 (0.02–0.07)		0.03 (0.02–0.09)		0.03 (0.02–0.06)		0.260
Second measurement	0.05 (0.03–0.10)		0.11 (0.08–0.30)		0.03 (0.02–0,0.04)		**<0.001** ^*^

The asterisk and bold values represent statistically significant results (*p* < 0.05).

A total of 80 patients with AIS were included, comprising 37 (46.3%) in the infection group and 43 (53.7%) in the non-infection group. The mean age was 62.4 ± 11.2 years, with patients in the infection group being significantly older than those in the non-infection group (66.8 vs. 58.7 years, *p* = 0.003). Most patients were aged ≥ 60 years (58.8%), and this age distribution differed significantly between groups (*p* = 0.005). Females were more common in the infection group (56.8% vs. 30.2%, *p* = 0.027). Obesity was the predominant BMI category (55.0%), followed by overweight (20.0%) and normal weight (22.5%), with a significant difference across groups (*p* = 0.029). Hypertension (62.5%), dyslipidaemia (73.8%), and diabetes mellitus (42.5%) were the most prevalent cardiovascular risk factors, with no significant intergroup differences. Dietary patterns showed high sodium, sugar, and fat intake in over 40% of patients. Most patients were admitted to non-intensive wards (96.3%). Multiple infarct lesions were more common than single lesions (75.0% vs. 16.3%). The median NIHSS score at admission was 4.6 (IQR 4.0–9.0), and mild stroke predominated (50.0%), followed by moderate stroke (41.3%). Dysphagia occurred more frequently in the infection group (29.7% vs. 14.0%, *p* = 0.041). Regarding interventions, nasogastric tube use was recorded in 17.5% of patients, while ventilator, central venous catheter, urinary catheter, total parenteral nutrition (TPN), blood transfusion, and tracheostomy were infrequent. TPN use was significantly higher in the infection group (13.5% vs. 0%, *p* = 0.013). The median white blood cell count at admission was 9.8 × 10^9^/L, with leukocytosis (>10,000) more common in the non-infection group (62.8% vs. 32.4%, *p* = 0.006). Platelet counts were largely within normal range in both groups. Median procalcitonin levels were low at first measurement in both groups, but at the second measurement were significantly higher in the infection group [0.11 (0.08–0.30) vs. 0.03 (0.02–0.04) ng/mL, *p* < 0.001].

In the initial logistic regression analysis (Table 2), several clinical factors were found to be associated with infection risk, including age, BMI, dyslipidemia, and elevated white blood cell counts. Older age significantly increased the risk of infection (OR = 4.97, 95% CI: 1.42–17.39, *p* = 0.011), while dyslipidemia also emerged as a strong predictor (OR = 8.46, 95% CI: 1.65–43.57, *p* = 0.009). Increased white blood cell count was significantly associated with infection (OR = 4.09, 95% CI: 1.18–14.13, *p* = 0.023), indicating an inflammatory response. However, after adjustment in the final multivariate model, traditional clinical factors lost their statistical significance, and several molecular biomarkers emerged as independent predictors of infection. Specifically, NMDAR (OR = 5.28 × 10^14^, *p* = 0.034), butyrate (OR = 2.18 × 10^15^, *p* = 0.004), TMAO (OR = 1.84 × 10^15^, *p* = 0.009), and RANKL (OR = 8.73 × 10^15^, *p* = 0.002) were significantly associated with increased infection risk.

**Table 2:** Risk factors for infection.

Variable	Initial model			Final model		
	OR	CI 95%	*p* value	OR	CI 95%	*p* value
Constanta				1.89		
Age	4.97	1.42–17.39	**0.011** ^*^	0.94	–	0.051
Gender	3.62	0.96–13.54	**0.050**	–	–	–
BMI	0.88	0.78–0.99	**0.041** ^*^	–	–	0.096
Dyslipidemia	8.46	1.65–43.57	**0.009**	–	–	–
High sugar diet	1.34	0.38–4.67	0.623	–	–	–
High fat diet	0.41	0.10–1.52	0.158	–	–	–
Itensive care unit	1.00 × 105	–	1.000	–	–	–
NIHSS score	1.09	0.99–1.23	0.079	–	–	–
Stroke severity	1.15	0.96–1.39	0.102	–	–	–
Dysphagia	3.98	0.77–20.52	0.158	–	–	–
Nasogastric tube (NGT)	0.19	0.02–1.73	0.134	–	–	–
Total parenteral nutrition (TPN)	1.00 × 105	–	1.000	–	–	–
White blood cells	4.09	1.18–14.13	**0.023** ^*^	–	–	–
Platelet count	2.17	0.41–11.36	0.350	–	–	–
Procalcitonin 2	3.05 × 10^15^	–	0.274	–	–	–
NMDAR	4.87 × 10^14^	–	1.000	5.45 × 10^14^	–	0.034^*^
Butyrate	1.69 × 10^15^	–	1.000	2.32 × 10^15^	–	0.004^*^
TMAO	3.65 × 10^6^	–	1.000	1.92 × 10^15^	–	0.009^*^
RANKL	6.91 × 10^15^	–	1.000	8.97 × 10^15^	–	0.002^*^
iFABP	9.31 × 10^4^	–	1.000	–	–	–
LPS	2.25 × 10^8^	–	1.000	–	–	–

Final model derived using stepwise backward logistic regression including variables with *p* < 0.10 in univariate analysis. The asterisk and bold values represent statistically significant results (*p* < 0.05).

### Diagnostic performance biomarker

The diagnostic performance analysis of biomarkers in detecting post-stroke infection among patients with acute ischemic stroke showed that NMDAR demonstrated the highest diagnostic accuracy with an AUC of 0.912 (95% CI: 0.846–0.978), a cut-off value of 3.10, sensitivity of 86.5%, and specificity of 90.7%, resulting in a Youden Index of 0.772 (Table 3). Butyrate also exhibited strong diagnostic value with an AUC of 0.867 (95% CI: 0.778–0.954), sensitivity of 83.8%, specificity of 88.4%, and a Youden Index of 0.768. Similarly, TMAO achieved an AUC of 0.864 (95% CI: 0.780–0.950), with sensitivity and specificity values of 86.5% and 88.4%, respectively, and a Youden Index of 0.749. RANKL obtained an AUC of 0.880 (95% CI: 0.802–0.958) and demonstrated perfect sensitivity (100%), although its specificity was lower at 65.1%, yielding a Youden Index of 0.651. Meanwhile, iFABP showed an AUC of 0.893 (95% CI: 0.825–0.962), with sensitivity of 89.2%, specificity of 76.7%, and a Youden Index of 0.659. LPS also demonstrated considerable diagnostic ability, with an AUC of 0.895 (95% CI: 0.823–0.968), sensitivity of 89.2%, specificity of 74.4%, and a Youden Index of 0.636. Collectively, these results indicate that all evaluated biomarkers have good potential in diagnosing post-stroke infection in acute ischemic stroke, with NMDAR showing the best overall performance.

**Table 3:** Diagnostic accuracy and cut-off values of biomarkers for post-stroke infection in acute ischemic stroke.

Biomarker	AUC	*p* value	CI 95%	Cut-off	Sensitivity	Specificity	Youden index
NMDAR	0.912	<0.001^*^	0.846–0.978	3.10	0.865	0.907	0.772
Butirat	0.867	<0.001^*^	0.778–0.954	10.02	0.838	0.884	0.768
TMAO	0.864	<0.001^*^	0.780–0.950	537.20	0.865	0.884	0.749
RANKL	0.880	<0.001^*^	0.802–0.958	7.80	1.00	0.651	0.651
iFABP	0.893	<0.001^*^	0.825–0.962	3.05	0.892	0.767	0.659
LPS	0.895	<0.001^*^	0.823–0.968	113.5	0.892	0.744	0.636

### Profil gut miikrobiota

**Figure 1:**
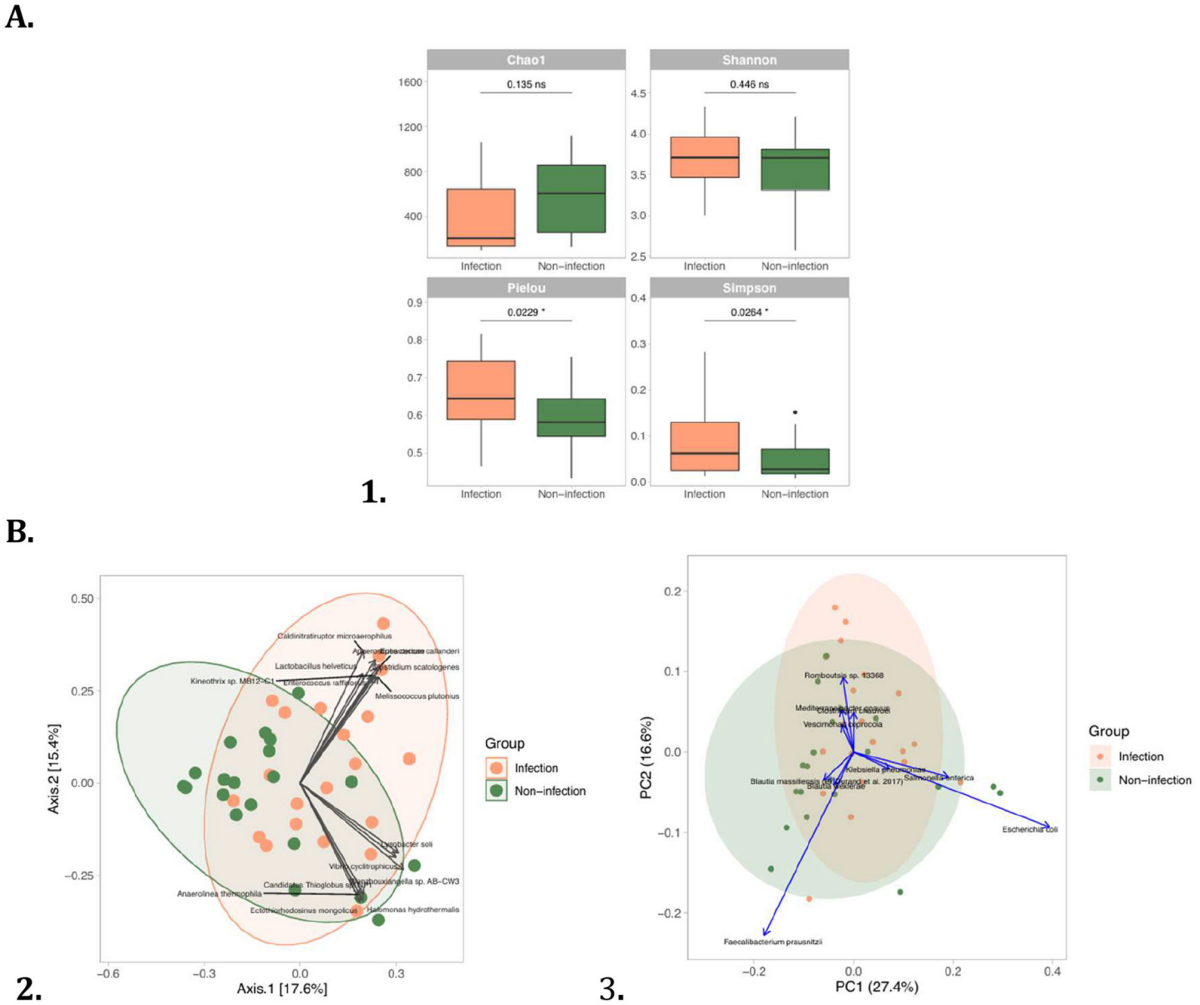
**(A)** Alpha diversity and **(B)** Beta diversity of gut microbiota in patients with acute ischemic stroke with and without bacterial infection. (1) Boxplots of alpha diversity indices (Chao1, Shannon, Pielou, and Simpson indices *p*<*0.05, Wilcoxon rank-sum test*); (2) PcoA biplot top 15 contributors gut microbiota; (3) PcoA biplot top 10 contributors species gut microbiota.

These findings were supported by alpha diversity analysis, which revealed no significant differences in species richness (Chao1 index) or overall diversity (Shannon index) between patients with acute ischemic stroke with and without bacterial infection (median Chao1: Infection 512.3 vs Non-infection 643.7, *p* = 0.135; median Shannon index: Infection 3.88 vs Non-infection 4.02, *p* = 0.446). However, significant reductions were observed in community evenness (Pielou's index: Infection 0.68 vs Non-infection 0.74, *p* = 0.0229) and diversity dominance balance (Simpson index: Infection 0.085 vs Non-infection 0.124, *p* = 0.0264), indicating a less even distribution of microbial taxa and increased dominance of certain species in the infection group (Figure 1A.1). Rarefaction curves confirmed adequate sequencing depth for both groups, as indicated by the clear plateau in observed species counts (Figure 1A.1).

Beta diversity analysis was performed to evaluate differences in gut microbial community composition between patients with acute ischemic stroke with and without bacterial infection. Principal Coordinates Analysis (PCoA) based on Bray–Curtis distances demonstrated a significant separation between the two groups (PERMANOVA: *R*^2^ = 0.057, *F* = 2.37, *p* = 0.005) (Figure 1B.2). The BETADISPER test indicated no significant difference in dispersion between groups (*p* = 0.241), confirming that the observed differences were due to shifts in community structure rather than variation in within-group dispersion. The top 15 taxa contributing to the observed variance included *Caldinitratoruptor microaerophilus, Anaerostipes caccae, Eubacterium callanderi, Wenzhouxiangella sp. AB-CW3*, and *Clostridium scatalogenes*, among others (Figure 1B.2). Several of these high-contribution taxa were enriched in the infection group, such as *Enterococcus raffinosus* and *Melissococcus plutonius*, whereas non-infection patients were more associated with taxa like *Kineothrix sp. MB12-C1* and *Halomonas hydrothermalis*. Similarly, Principal Component Analysis (PCA) further highlighted the microbial taxa driving the separation between groups (Figure 1B.3). The top 10 contributing species included *Escherichia coli, Faecalibacterium prausnitzii, Salmonella enterica, Romboutsia sp. 13368* (9.70%), and *Mediterraneibacter gnavus* (3.31%) (Figure 1B.3). Notably, *Escherichia coli* and *Salmonella enterica* were predominantly associated with the infection group, while *Faecalibacterium prausnitzii* a known anti-inflammatory commensal was more abundant in the non-infection group.

**Figure 2:**
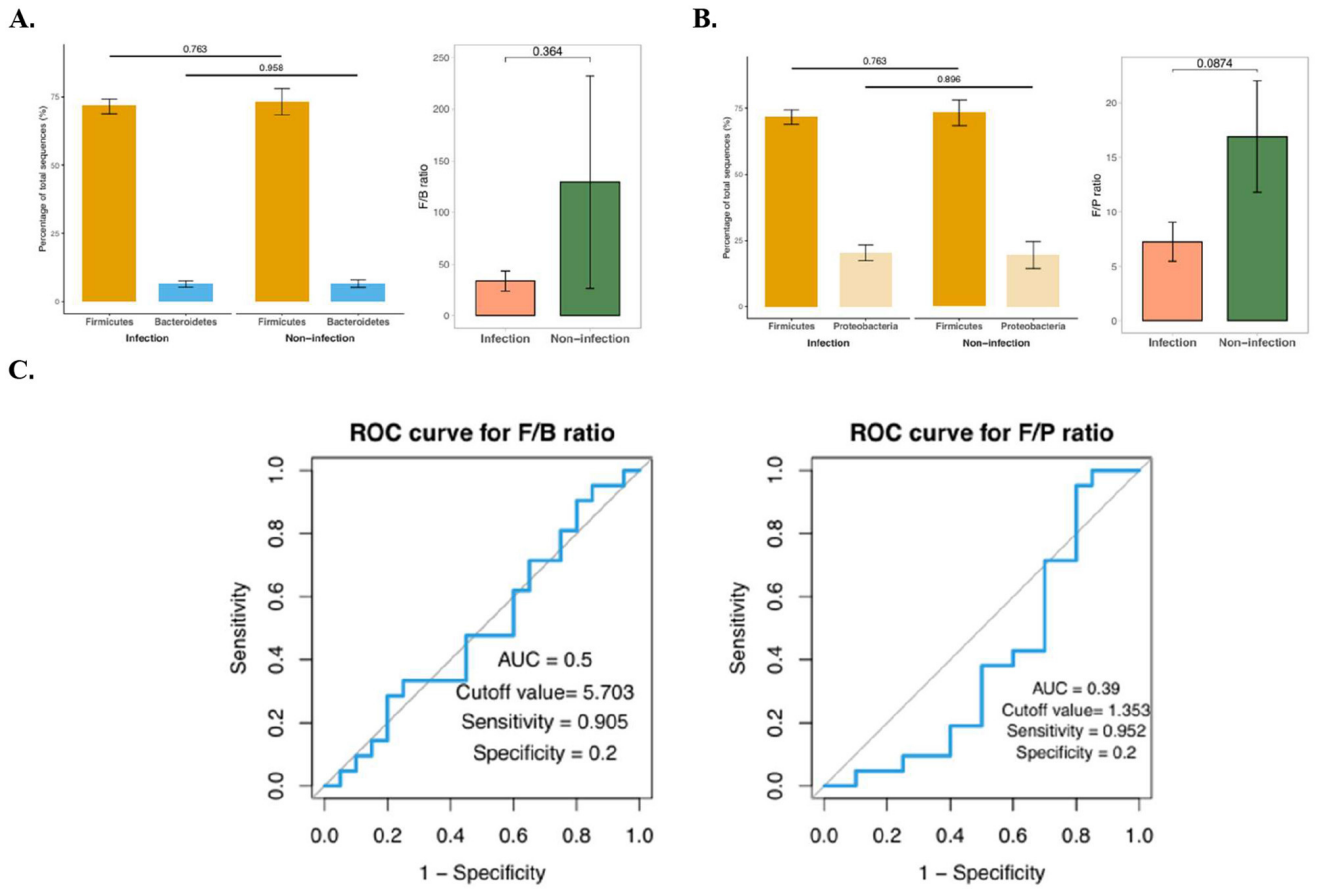
Comparison of gut microbiota composition and diagnostic performance of microbial ratios in patients with and without infection. **(A)** Firmicutes-to-Bacteroidetes (F/B) ratio; **(B)** Firmicutes-to-Proteobacteria (F/P) ratio; **(C)** Receiver operating characteristic (ROC) curves showing the discriminatory performance of F/B and F/P ratios for infection status.

The relative abundance of dominant phyla was compared between infection and non-infection groups (Figures 2A, B). Firmicutes constituted the majority in both groups, whereas Bacteroidetes and Proteobacteria were present in lower proportions. Although no statistically significant differences were observed in the relative abundances of Firmicutes vs. Bacteroidetes or Proteobacteria (*p* > 0.05), trends were noted in the calculated ratios. The F/B ratio was higher in the non-infection group compared with the infection group (median ratio ~130 vs ~30, respectively), although the difference did not reach statistical significance (*p* = 0.364). Similarly, the F/P ratio tended to be higher in the non-infection group compared with the infection group (median ratio ~17 vs ~7, *p* = 0.0874), suggesting a shift in microbial balance during infection toward greater representation of Proteobacteria, a phylum often linked to dysbiosis and inflammation.

Receiver operating characteristic (ROC) analysis was used to evaluate the discriminative ability of F/B and F/P ratios for infection status (Figure 2C). The F/B ratio showed an area under the curve (AUC) of 0.50, with a cutoff value of 5.703, sensitivity of 90.5%, but low specificity of 20%, indicating limited predictive power. The F/P ratio performed slightly worse, with an AUC of 0.39, cutoff value of 1.353, sensitivity of 95.2%, and specificity of 20%. Although both ratios (F/B and F/P) showed a trend toward reduction in infection cases, reflecting dysbiosis and expansion of Proteobacteria, their ROC performance suggests they are not reliable standalone biomarkers for distinguishing infection from non-infection. Instead, these ratios may be more useful when combined with other microbial signatures or clinical parameters. Because the AUC values of the F/B (0.50) and F/P (0.39) ratios were non-informative, we avoided drawing biological conclusions from these findings and did not interpret them as clinically relevant diagnostic markers.

**Figure 3:**
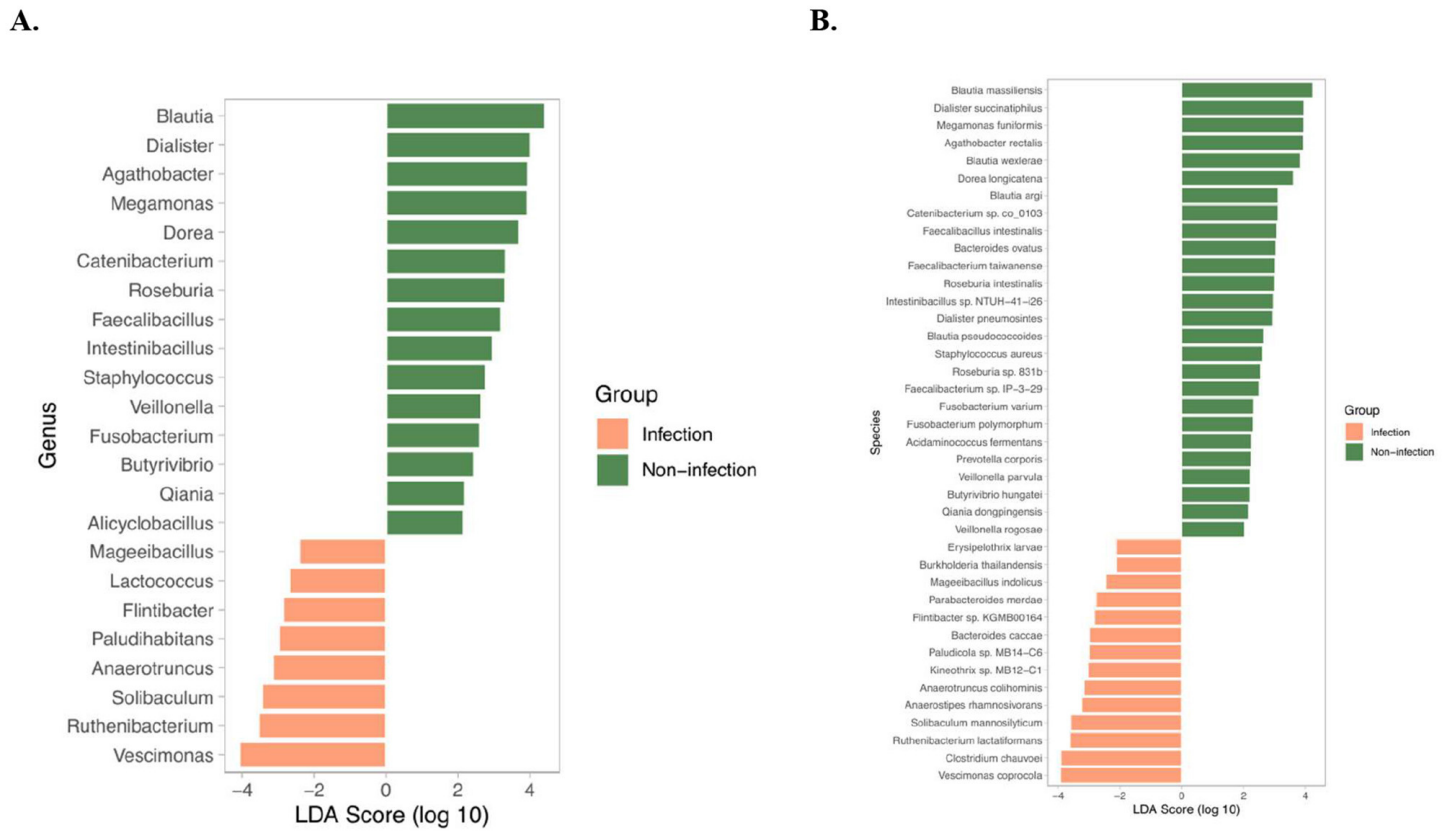
Differently Abudant taxa identified by LEfSe. LDA bar plots show significantly enriched species across groups based on LEfSe analysis (LDA score > 2.0, *p* < 0.05). **(A)** Genus level; **(B)** species level.

LEfSe analysis was performed to identify bacterial taxa that were differentially enriched between infection and non-infection groups (Figures 3A, B). At the genus level (Figure 3A), several commensal and short-chain fatty acid (SCFA)-producing taxa were significantly enriched in the non-infection group, including Blautia, Dialister, Agathobacter, Megamonas, Dorea, Roseburia, Faecalibacillus, and Catenibacterium. These taxa are well recognized for their roles in maintaining intestinal homeostasis, SCFA production, and anti-inflammatory activity. In contrast, the infection group was enriched in opportunistic or potentially pathogenic genera such as Vescimonas, Ruthenibacterium, Solibaculum, Anaerotruncus, and Lactococcus, which have been associated with gut dysbiosis and systemic inflammatory conditions. At the species level (Figure 3B), beneficial commensals including Blautia massiliensis, Dialister succinatiphilus, Agathobacter rectalis, Dorea longicatena, Faecalibacterium taiwanense, and Roseburia intestinalis were significantly enriched in the non-infection group. Conversely, the infection group displayed higher abundance of opportunistic and pathogenic species such as Vescimonas coprocola, Clostridium chauvoei, Ruthenibacterium lactatiformans, Anaerotruncus colihominis, Mageeibacillus indolicus, Parabacteroides merdae, and Burkholderia thailandensis. Many of these species have been previously linked to pro-inflammatory states, impaired gut barrier function, and systemic infections.

LEfSe analysis clearly distinguished the microbial signatures of infection vs. non-infection. The non-infection group was dominated by beneficial commensals with SCFA-producing capacity, reflecting a protective microbial profile. In contrast, the infection group harbored higher levels of opportunistic and pathogenic bacteria that may contribute to gut barrier disruption, systemic inflammation, and infection-related complications. These findings strengthen the evidence that microbial dysbiosis in infection is characterized not only by loss of beneficial taxa but also by expansion of pathogenic lineages.

## Discussion

Because this was an observational study, our findings indicate statistical associations rather than causal relationships. Therefore, terms such as ‘predict' or ‘improve diagnostic accuracy' are used in a statistical sense and do not imply biological causality. This study clearly demonstrates that integrating circulating biomarkers with gut microbiota profiling significantly enhances diagnostic accuracy for PSI in AIS patients. ROC analysis revealed that NMDAR achieved the highest diagnostic performance (AUC 0.911; sensitivity 86.5%; specificity 90.7%), followed by iFABP (AUC 0.894), LPS (AUC 0.896), butyrate (AUC 0.866), TMAO (AUC 0.865), and RANKL (AUC 0.881). These biomarkers reflect key pathological processes: immune activation (NMDAR, RANKL), gut barrier disruption (iFABP), bacterial translocation/endotoxemia (LPS), and microbial metabolite dysregulation (butyrate, TMAO). Previous studies support these findings: elevated iFABP levels indicate intestinal injury in stroke; increased plasma LPS is linked to poor outcomes in AIS; higher TMAO correlates with worse stroke prognosis; and depletion of butyrate-producing taxa increases vulnerability to infection post-stroke. Although direct data on RANKL in PSI are limited, RANK/RANKL signaling is recognized in CNS inflammation and may serve as a biomarker in neurological injury (7–11).

These results are anchored in the gut–brain–immune axis framework. AIS triggers immune suppression, autonomic dysregulation, and disruption of the gut barrier, facilitating bacterial translocation, endotoxemia, and systemic inflammation. In our microbiota profiling, non-infected patients were enriched with SCFA-producing taxa like *Faecalibacterium prausnitzii* and *Roseburia intestinalis*, which support gut integrity and mitigate inflammation. In contrast, infected patients showed overrepresentation of opportunistic pathogens such as *Escherichia coli* and *Salmonella enterica*. This dysbiotic signature aligns with Xia et al. (3), who identified depletion of *Roseburia* and opportunistic pathogen enrichment as independent predictors of PSI. Furthermore, Li et al. (1) demonstrated that predictive models combining clinical variables and microbiota features achieved higher AUC (~0.81) than models using either alone.

Mechanistically, the enrichment of opportunistic pathogens (e.g., *E. coli, Salmonella enterica*) and depletion of SCFA-producing commensals (e.g., *Faecalibacterium prausnitzii, Roseburia intestinalis*) may contribute to mucosal barrier weakening, allowing for bacterial translocation reflected by elevated LPS and iFABP levels. Concurrent increases in neuroimmune markers such as NMDAR and RANKL suggest a systemic inflammatory response linking dysbiosis to PSI risk. These integrated findings expand upon prior studies by demonstrating a combined biomarker–microbiota axis that offers higher discriminative value than either domain alone.

Biological alterations following stroke such as dysbiosis, bacterial translocation, mucosal injury, and microbial metabolite changes are more influential predictors of PSI than conventional clinical risk factors. For instance, elevated iFABP indicates intestinal mucosal damage and bacterial translocation (5). While high LPS levels confirm endotoxemia, which worsens stroke prognosis (4). Elevated TMAO supports recent meta-analyses linking this metabolite to vascular inflammation and poor stroke outcomes (6). Conversely, decreased butyrate production due to loss of SCFA-producing bacteria impairs immune regulation and gut homeostasis (3). Taken together, the integration of circulating biomarkers and microbiota signatures provides a comprehensive understanding of PSI pathogenesis. From a clinical standpoint, these findings suggest that biomarker thresholds identified in this study (e.g., NMDAR >3.12, iFABP >3.07 ng/mL, and LPS >113.6 pg/mL) could assist clinicians in stratifying PSI risk within the first week after stroke. High-risk patients could benefit from intensified monitoring, early microbiological screening, or prophylactic interventions such as swallowing therapy and infection control protocols. Moreover, integrating microbiota profiling into predictive algorithms may enable individualized preventive strategies, especially for patients showing dysbiosis signatures before overt infection occurs. Thus, our proposed combined model provides a practical framework for early PSI prediction and management. Consistent with previous reports, pneumonia represented the predominant infection type and was associated with greater stroke severity and reduced microbial diversity. Although mortality was low in our cohort, all fatal cases occurred among infected patients, underscoring the clinical relevance of early PSI identification.

The gut–brain axis is a bidirectional communication system linking the central nervous system and the gastrointestinal tract through neural, immune, neuroendocrine, and metabolic pathways. After an acute ischemic stroke, gut–brain axis dysfunction emerges as a pivotal mechanism impacting stroke outcomes. Stroke triggers autonomic dysregulation and activation of the hypothalamic–pituitary–adrenal (HPA) axis, leading to immunosuppression and gut motility alterations. Consequently, gut dysbiosis develops, characterized by overgrowth of Enterobacteriaceae and loss of beneficial microbes, which worsens systemic inflammation and infarct severity, as shown in both human and animal models (12–14).

Recent reviews consolidate this mechanistic framework: Sheng-Yu Zhou et al. (15) describe how ischemic stroke impairs gut motility, disrupts barrier integrity, and alters microbial composition, contributing to poor prognosis. The bidirectional nature of this interaction implies that not only does stroke affect the gut, but these gut alterations feedback to aggravate brain injury through immune and metabolic pathways (13).

Emerging literature emphasizes the role of the microbiota–gut–brain axis (MGBA) in modulating neuroinflammation. A 2025 review in *Biomolecules* highlights how gut microbiome-derived signals regulate microglial and astrocyte responses, influencing neuroinflammatory dynamics after ischemic stroke, and underscores the potential for MGBA-targeted therapies including dietary interventions and probiotics (14, 16–18).

## Clinical implications

Our findings suggest that circulating biomarkers such as NMDAR, iFABP, and LPS could be integrated into early-risk stratification workflows for PSI within the first 72 h of AIS. These biomarkers can be measured using widely available ELISA or LC-MS/MS assays, with relatively low cost compared to metagenomic analysis. Gut microbiota profiling, although currently more costly, could be incorporated in high-risk patients or research settings to identify dysbiosis signatures before infection onset. Future validation studies should evaluate feasibility, cost-effectiveness, and development of point-of-care platforms for rapid biomarker assessment.

## Limitations

Despite these promising results, this study has several limitations. First, the follow-up period was restricted to seven days, while PSI may develop within 2 weeks or longer after stroke onset, meaning some late-onset cases may have been missed. Second, microbiota profiling was conducted using 16S rRNA sequencing, which provides taxonomic resolution but lacks functional insights and may not detect minority species compared with shotgun metagenomics. Potential confounders such as antibiotic or probiotic use and nutritional interventions were not comprehensively assessed, although these factors may influence both microbiota composition and infection risk. The sample size of this study (37 infected vs. 43 non-infected patients) provided adequate statistical power for ROC analysis; however, the imbalance between groups may reduce the stability of the multivariate regression model. With a relatively small number of infected cases, the wide confidence intervals observed in several predictors should be interpreted with caution. Larger, multicenter studies with more balanced case distribution are needed to validate the robustness of the combined biomarker–microbiota model. Another limitation is the use of 16S rRNA sequencing restricted to the V3–V4 region, which provides limited taxonomic resolution and does not allow functional profiling. Shotgun metagenomic sequencing was not performed due to cost constraints and specimen availability at the time of study. Although descriptive shifts in F/B and F/P ratios were observed, these findings did not reach statistical significance and showed poor discriminative ability; thus, no mechanistic interpretation is warranted. Future studies should incorporate metagenomics or functional prediction tools such as PICRUSt or Tax4Fun to better elucidate mechanistic pathways linking dysbiosis and PSI.

## Conclusion

This study demonstrates that integrating circulating biomarkers with gut microbiota profiling provides superior diagnostic accuracy in predicting post-stroke infection (PSI) among patients with acute ischemic stroke (AIS). Biomarkers such as NMDAR, iFABP, LPS, TMAO, butyrate, and RANKL reflect key mechanisms including immune activation, gut barrier disruption, bacterial translocation, and microbial metabolite imbalance, while gut dysbiosis characterized by depletion of SCFA-producing taxa and enrichment of opportunistic pathogens further amplifies infection risk. These findings highlight the pivotal role of the gut–brain–immune axis in PSI pathogenesis and suggest that combined biomarker–microbiota models may enable earlier risk stratification and targeted preventive interventions in acute stroke care. Future multicenter studies with larger cohorts and advanced sequencing methods are warranted to validate these results and explore microbiota-targeted therapeutic strategies.

## Data Availability

The datasets associated with this study have been deposited in a public repository and are available at the following link: https://doi.org/10.5281/zenodo.18946959.
